# Perioperative Ketorolac Use and Its Association with Opioid Consumption in Facelift Surgery: a Retrospective Cohort Study

**DOI:** 10.1093/asjof/ojag138.013

**Published:** 2026-07-24

**Authors:** W Nicholas Jungbauer, Tanner Carcione, Jonathan L Jeger, Ashley L Howarth, Terri R Maffi

## Abstract

**Introduction:**

Facelift surgery has remained a popular aesthetic procedure in the United States in recent years as patients seek to maintain a youthful appearance.¹ An important consideration in the context of facelift surgery is the management of post-operative pain,²^,^³ both to enhance the patient experience and to reduce the risk of pain-induced hypertension and tachycardia, which are associated with hematoma formation. An expanding hematoma poses a substantial risk to the peri-operative facelift patient due to the possibility of airway compression,⁴ and surgeons take extensive measures to prevent this complication. Additionally, hematoma formation may also adversely affect the aesthetic outcome of the procedure. Previous studies have evaluated the effect of nonsteroidal anti-inflammatory drugs (NSAIDs) in the context of peri-operative pain in facial plastic surgery,⁵ but there is a paucity of data concerning the use of intravenous ketorolac and its impact on post-operative pain control in facelift patients. The objective of this study is to compare post-operative narcotic use in facelift patients with and without ketorolac supplementation, with the goal of evaluating its efficacy in pain control relative to its potential risk of hematoma formation.

**Methods:**

A retrospective chart review was performed to identify patients who underwent facelift by a single surgeon (T.M.) between 2019 and 2025. All facelift patients were admitted for one night of post-operative monitoring, and peri-operative pain medication administration was evaluated. Patients were treated with one of two protocols: a regimen that employed scheduled ketorolac 15 mg every 6 hours post-operatively with narcotic supplementation as needed, or a regimen that did not include scheduled ketorolac and instead used multimodal pain management such as acetaminophen and oxycodone. Both cohorts were treated with benzodiazepines (diazepam) as needed. Demographic variables and concomitant procedures were extracted into a standardized Microsoft Excel worksheet. Primary outcome variables included peri-operative morphine milligram equivalents (MME), benzodiazepine use, and hematoma rate. Data were assessed for normality, and univariate analysis was performed in SPSS (IBM, Version 26) using the Fisher’s Exact test for categorical variables and Mann-Whitney U test for continuous variables.

**Results:**

In total, 101 patients met inclusion criteria for the study, including 95 females (91.4%) with a mean ± SD age of 62.9 ± 14.1 years. Sixty-four patients (63.4%) were treated with the ketorolac protocol, and 37 patients (36.6%) were treated without ketorolac. In the ketorolac cohort, patients received a mean ± SD of 21.1 ± 22.0 MME of narcotics in the peri-operative period, compared to 29.6 ± 31.3 MME in the non-ketorolac cohort (p = 0.394) (Figure 1). Patients treated with ketorolac received a mean ± SD of 11.6 ± 10.2 mg of diazepam post-operatively, compared to 11.6 ± 8.3 mg in the non-ketorolac protocol (p = 0.773). The hematoma rate was 1.6% in the ketorolac cohort and 6.3% in the non-ketorolac cohort (p = 0.55). No patients were admitted for longer than two nights post-operatively.

**Conclusion:**

Administration of scheduled post-operative ketorolac was not associated with a decrease in total narcotic use in the peri-operative period in this cohort of 101 facelift patients. The overall narcotic use in both groups was modest, suggesting that modern multimodal analgesia achieves adequate pain control in facelift patients. Furthermore, ketorolac did not have a statistically significant effect on hematoma rate. These data suggest that patients undergoing facelift may not benefit from the administration of scheduled ketorolac in terms of total narcotic requirements, as both cohorts used a moderate amount of narcotics regardless of ketorolac administration. Given the theoretical risk of NSAID use and post-operative bleeding, as well as the devastating consequences of post-facelift hematoma, surgeons should consider whether the use of ketorolac in these patients aligns with their peri-operative pain control goals.
